# A population-based study on the seroepidemiology of varicella zoster virus among children and adolescents in Fujian, China

**DOI:** 10.1186/s12879-026-13917-z

**Published:** 2026-08-06

**Authors:** Dong Li, Zhifei Chen, Hairong Zhang, Suhan Zhang, Mengping Zhang, Xiuhui Yang, Xiaoling Chu, Dongjuan Zhang

**Affiliations:** Fujian Provincial Key Laboratory of Zoonosis Research, Fujian Center for Disease Control and Prevention, Fuzhou, 350001 China

**Keywords:** Seroepidemiological, Varicella-zoster virus, GMC, Vaccine

## Abstract

**Background:**

Varicella continues to pose a significant public health challenge for children and adolescents in China. Although vaccination is available, the disease remains highly prevalent among children in Fujian. This study aimed to evaluate the seroepidemiology of varicella-zoster virus (VZV) and to explore the factors contributing to its high incidence in individuals aged 0–17 years.

**Methods:**

A cross-sectional serosurvey was conducted in 2023. Serum samples from 2,675 eligible individuals were tested for VZV IgG using ELISA. Demographic and vaccination data were collected via questionnaire.

**Results:**

A total of 2,675 participants, aged between 2 days and 17 years with a median age of 6.5 years, were included. The overall seropositivity rate of VZV IgG was 47.7% (95% CI: 45.5–49.3), with a geometric mean concentration (GMC) of 81.3 mIU/mL (95% CI: 77.0–85.9). Marked variations in these immunity markers were observed across age groups, genders, and vaccination statuses. Notably, varicella vaccination coverage was substantially low: only 54.9% of children aged 1–17 years had received one dose, and merely 17.4% were fully vaccinated with two doses. A clear post-vaccination antibody waning pattern was also evident. Both seropositivity rate and GMC began to decline one year after vaccination, decreasing by more than 50% after four years.

**Conclusions:**

The seropositivity rates and GMC of varicella antibodies were low among individuals aged 0–17 years in Fujian Province, and vaccination coverage was insufficient, particularly for the second dose (< 20%). Antibody titers declined markedly over time, which may be partly associated with the high incidence of varicella in this region. To ensure the continuity of surveillance data, further efforts are needed to strengthen epidemiological surveillance of varicella in younger age groups and to promote the timeliness and completeness of vaccination.

**Clinical trial number:**

Not applicable.

## Introduction

Varicella, caused by the varicella-zoster virus (VZV), is a systemically infectious disease characterized by vesicular eruptions [1, 2]. While typically self-limiting, clinical severity can escalate to severe complications including pneumonia, encephalitis, and congenital varicella syndrome, with case-fatality rates disproportionately affecting neonates, pregnant women, geriatric populations, and immunocompromised hosts [3]. Varicella mainly affected the younger age groups; 52–78% of incident cases occurred in children < 6 years of age, and 89–95.9% of cases occurred before adolescence [4]. In the World Health Organization’s position paper published in 2014, it was emphasized that without a vaccination program, the globally prevalent varicella virus would infect the majority of individuals during early adulthood, with notable geographical differences in the age of varicella infection [5]. In temperate, high-income countries during the pre-vaccination era, over 90% of infections occurred before adolescence, and less than 5% of adults remained susceptible. In many tropical settings, infection tends to be acquired at older ages, resulting in higher susceptibility among young adults [5]. Furthermore, prior to the introduction of vaccination, the recorded epidemic cycles of chickenpox occurred approximately every 2 to 5 years [3].

Between 2007 and 2021, a total of 10,446 public health events or outbreaks related to varicella were recorded in China, with a significant concentration in educational institutions; 90.0% of these cases occurred in primary and secondary schools and kindergartens [6, 7]. The epidemiological trends in Fujian Province from 2019 to 2024 reveal an age-stratified incidence pattern. The mean annual incidence reached 33.50 per 100,000 population, peaking at 133.0-179.8 per 100,000 among preschoolers aged 4 to 6 years (Fig. 1A). Notably, in recent years, the incidence of varicella has gradually shifted toward older age groups. In 2024, the incidence among individuals aged 15 to 19 years in Fujian Province was significantly higher than in previous years, reaching 193.0 per 100,000 (Fig. 1B). Immunoprophylaxis through the live-attenuated varicella vaccine (VarV) remains the cornerstone of prevention. Compelling evidence from municipalities with universal VarV implementation, such as Beijing, Tianjin, and Shanghai, indicates that two-dose regimens confer an efficacy of 92.3–98.7% in protection, significantly reducing outbreak frequencies [2]. In contrast, in Fujian Province, although children are recommended to receive one dose of the varicella vaccine at 12–24 months of age and another dose at 3–4 years of age, the lack of integration of VarV into the routine immunization program has resulted in suboptimal coverage and persistent epidemiological burdens. Additionally, gaps in serosurveillance complicate risk assessment: no population-level IgG seropositivity rates data have been collected since 2004, and vaccination coverage estimates remain fragmented.

In this context, it is crucial to obtain an update on the prevalence of varicella-susceptible individuals and to assess the impact of current vaccination interventions. To achieve this, a seroepidemiological survey was conducted involving a representative sample of the population aged 0 to 17 years in Fujian Province.

**Fig. 1:**
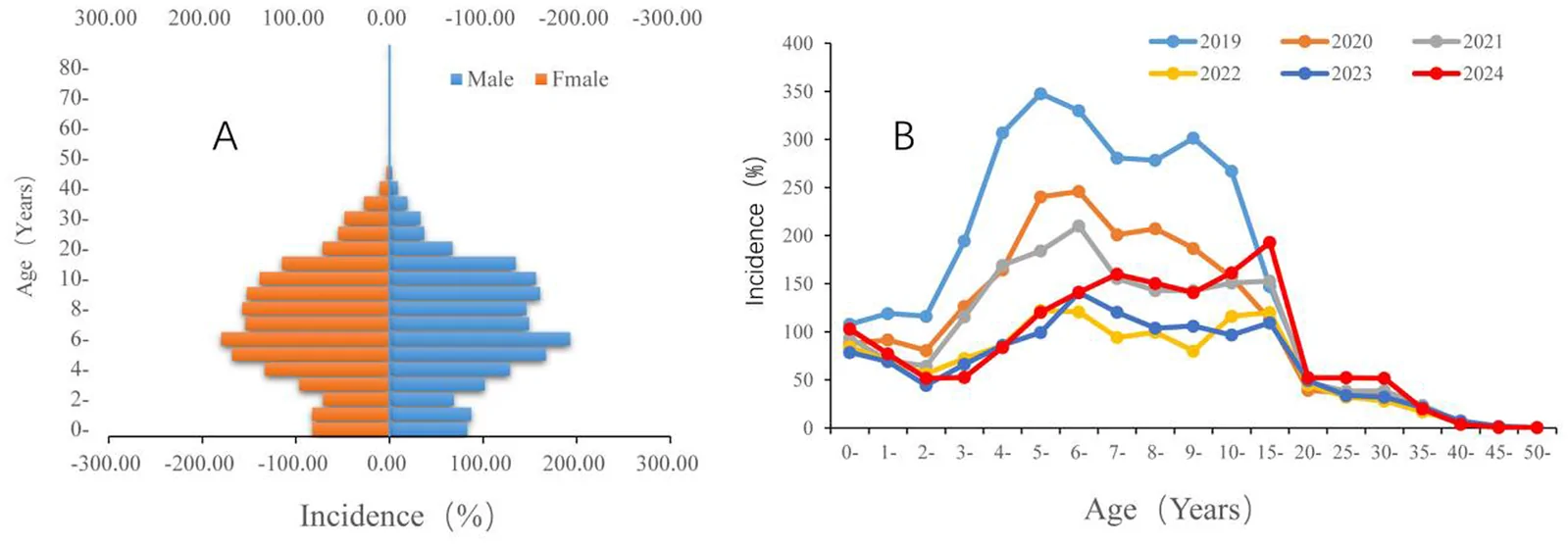
Incidence of varicella in different age groups in Fujian province, 2019–2024

## Materials and methods

### Serological survey

To ensure sample representativeness, a large-scale, population-based cross-sectional survey was conducted throughout Fujian province from March to June 2023 [8]. Initially, one county or district was randomly selected from each of the nine prefectures in the province as research sites. Subsequently, one township within each selected county or district were chosen using random number tables. Participants were recruited from individuals who had resided in the study area for more than three months, were in good health (defined as having an axillary temperature below 38℃ and no acute or chronic diseases), and who provided guardian or personal consent for blood sampling and completed informed consent forms. All subjects were stratified into nine age groups (0-, 1-, 2-, 3-, 4-, 5-, 6-, 12-, 15–17 years). Each subject provided 2-3 ml of venous blood and completed a questionnaire regarding personal information and immunization history, which included details such as age, gender, date of birth, inoculation status, dose received, and blood sampling dates. It is important to emphasize that persons for whom vaccination records are not available are considered unvaccinated.

### Laboratory test

After centrifuging the blood sample at 3,000 r/min for 10 min, serum samples were obtained and stored at -20 °C for further analysis. The Enzyme-linked Immunosorbent Assay (ELISA) method was used to detect antibodies to VZV (IgG anti-VZV) in the laboratory of the Fujian Center for Disease Control and Prevention (FJCDC). All tests were conducted by the same staff using a commercial ELISA kit (batch number: ESR104G) from Virion\Serion Technology GMBH, Germany. ELISA results were initially expressed as optical density (OD) at 405 nm and later converted to antibody concentration (mIU/ml) using SERION software, following kit instructions. According to the kit instructions, the serum anti-body concentration ranged from 15 to 2000 mIU/ml, the samples with a concentration > 2,000 mIU/ml were labeled as 2,000 mIU/ml, and those < 15 mIU/ml as 15 mIU/ml. Concentrations of ≥ 100 mIU/ml were considered positive, < 50 mIU/ml negative, and equivocal results (50–100 mIU/ml) were retested. Samples that remained equivocal upon retesting were classified as negative.

### Statistical analysis

EpiData software was used to input questionnaire data in duplicate. Seropositivity rates, geometric mean concentrations (GMC), and 95% confidence intervals (95% CIs) of IgG anti-VZV were calculated based on region, gender, and age. *P*-values < 0.05 were considered statistically significant. Significant differences in seropositivity rates and GMC by age, gender and vaccine doses were evaluated using Pearson’s *χ²* and non-parametric tests, with a significance level of 0.05. SPSS 20.0 was used for all analyses.

## Results

### Characteristics of the subjects

A total of 2,675 participants, aged between 2 days and 17 years with a median age of 6.5 years, were included in the study. The gender distribution was a ratio of 1.19:1.0 (1,453 males and 1,222 females).

### Sero-survey of varicella in 2023

The survey results (Table 1) indicated the overall seropositivity rates among healthy individuals aged 0–17 years in Fujian Province was 47.7% (95% CI: 45.5–49.3). A pronounced difference in seropositivity rates was observed across age groups (*P* < 0.001). The lowest seropositivity rates (12.7%; 95% CI: 8.7–16.8) was found in children under 1 year of age, followed by a sharp increase to 55.8% (95% CI: 50.0–61.7) by 3 years of age. Beyond this age, seropositivity rates exhibited a declining trend (χ2 = 4.5, *p* = 0.035), reaching its lowest level (43.9%) in the 12–14 year age group. However, a subsequent increase was observed in adolescents aged 15–17 years, with seropositivity rates rising to 50.8% (95% CI: 45.2–56.4).

The GMC of VZV IgG among healthy individuals aged 0–17 years in Fujian Province was 81.3 mIU/mL (95% CI: 77.0–85.9), with marked variation across age groups (*P* < 0.001). The age-related trend in GMC mirrored that of seropositivity rates. The lowest GMC was observed in infants < 1 year old (30.8 mIU/mL), followed by a rise to 48.2 mIU/mL in 1-year-olds. A further increase was noted in 2-year-olds (86.6 mIU/mL), after which GMC remained relatively stable, fluctuating between 97.0 and 115.5 mIU/mL in children aged 3–14 years.

The seropositivity rates was 42.8% (95% CI: 40.3–45.4) in males and 46.4% (95% CI: 43.6–49.2) in females. The GMCs were 79.6 mIU/mL (95% CI: 74.2–85.3) for males and 90.2 mIU/mL (95% CI: 83.3–93.7) for females. Although no marked gender difference was observed in seropositivity rates, GMC were remarked higher in females (*P* = 0.013).

**Table 1:** Distribution of anti-VZV IgG seropositivity rates and GMC in different age, gender and vaccination status in population aged 1–17 years in Fujian province in 2023 (*n* = 2,675)

Characteristics	Sample size(*n* = 2675)	Seropositivity rates *n*, %(95% CI)	*P* value	GMC(mIU/mL), (95% CI)	*P* value
**Age**					
0–12 moths	259	33,12.7%(8.7–16.8)	*χ2* = 181.3, *p*<0.001	30.8(27.1–35.5)	*H* = 474.9, *p*<0.001
1-years	299	81,27.1%(22.1–32.1)		48.2(41.8–56.1)	
2-years	281	139,49.5%(43.6–55.3)		86.6(74.0-101.0)	
3-years	274	153,55.8%(50.0-61.7)		111.1(95.8-129.1)	
4-years	295	158,53.6%(47.9–59.3)		97.0(83.3-113.3)	
5-years	299	152,50.8%(45.2–56.5)		104.9(92.1-120.5)	
6-years	371	190,51.2%(46.1–56.3)		115.5(102-130.1)	
12-years	294	129,43.9%(38.2–49.6)		91.8(78.7-108.1)	
15–17 years	303	154,50.8%(45.2–56.4)		115.4(97.6–137.0)	
**Gender**					
Boy	1453	622,42.8%(40.3–45.4)	*χ2* = 2.579, *p* = 0.108	79.6(74.2–85.3)	z=-2.486, *P* = 0.013
Girl	1222	567,46.4%(43.6–49.2)		90.2(83.3–93.7)	
**Number of vaccine doses**					
Zero	726	134,18.5%(15.6–21.3)	χ2 = 114.7, *p*<0.001	34.6(31.5–38.6)	*H* = 609.8, *P*<0.001
One	1326	666,50.2%(47.5–52.9)		105.9(99.6-113.1)	
Two	421	318,75.5%(71.4–79.6)		209.6(189.3-232.3)	
Unknown^#^	202	71,35.1%(28.6–41.7)		70.0(57.7–84.2)	
**Total**	2675	1268,47.7%(45.5–49.3)		81.3(77.0-85.9)	

#Unknown vaccination status (e.g. vaccinated but unable to trace, unclear recollection, etc.)

Among the 2,675 children aged 0–17 years, 726 had not received the varicella vaccine (VarV), including 259 infants under 1 year of age (ineligible for vaccination). Additionally, 202 children had an unknown vaccination status, while 1,747 had been vaccinated. Of the vaccinated group, 1,326 received one dose of VarV and 421 received two doses (Fig. 2).

Pronounced differences were observed in both seropositivity rates and GMC across groups with different vaccination histories (*P* < 0.001) (Table 1). Specifically, the vaccinated group exhibited remarkable higher seropositivity rates and GMC than the unvaccinated group (*P* < 0.001). Furthermore, individuals who received two doses had marked higher seropositivity rates and GMC compared to those who received one dose (*P* < 0.001). In contrast, those with unknown vaccination status showed lower immunity levels, with a seropositivity rates of 35.1% and a GMC of 70.0 mIU/mL.

**Fig. 2:**
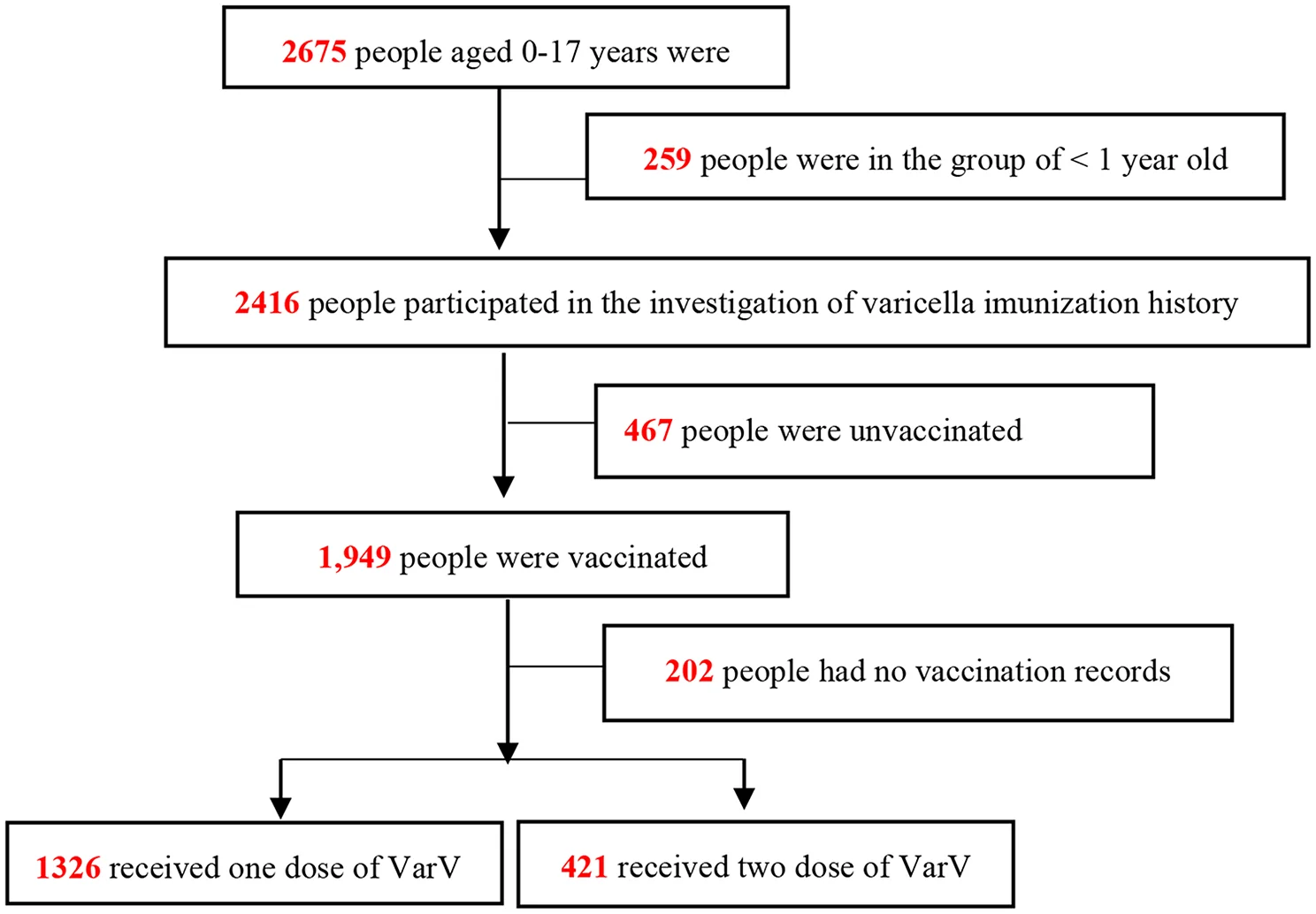
Investigation of immunization history in 0–17 year old population

### Vaccination coverage and attenuation of antibody levels

Through the Fujian Provincial Immunization Program Information System, we retrieved vaccination records for 1,949 individuals aged 1–17 years to obtain specific vaccination timings. The results showed that 202 individuals (10.4%) had no vaccination records in the system, while 1,747 (89.6%) had documented vaccination dates. The estimated varicella vaccination coverage rate in the 1–17 age group was 72.3% (1,747/2,416), with single-dose coverage at 54.9% (1,326/2,416) and two-dose coverage at 17.4% (421/2,416) (Fig. 2). An age-specific analysis of the 1,747 individuals with documented vaccination times revealed that 82.4% (1,440/1,747) received their first varicella vaccine dose at age 1. The second dose was primarily administered at ages 3 (26.1%, 112/421) and 4 (33.5%, 141/421) years (Fig. 3).

**Fig. 3:**
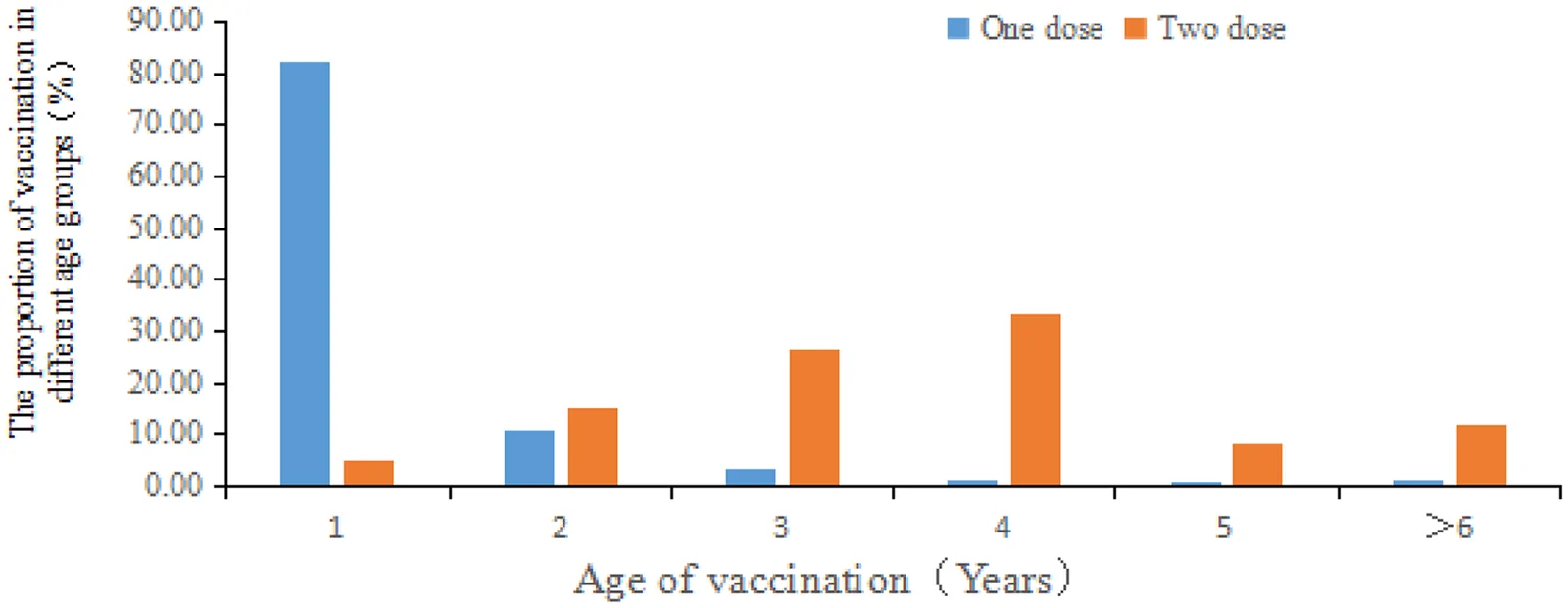
Age distribution of VarV vaccination in 1–17 year old population in Fujian

By calculating the interval between the time of vaccination and the time of sampling, we analyzed the changes in seropositivity rates and GMC at different intervals after vaccination in individuals who received only one dose of the varicella vaccine (1326 individuals) and those who received two doses (421 individuals). As illustrated in Fig. 4, remarkable differences were observed in the changes in seropositivity rates and GMC at various time intervals post-vaccination for children who received either one or two doses of the varicella vaccine (*P* < 0.001).Following a single dose of the varicella vaccine, the seropositivity rates and GMC among children increased to 41.6% and 95.1 mIU/mL, respectively, within six months. Subsequently, both metrics continued to rise, reaching 62.8% and 135.8 mIU/mL, and maintained these levels for two years. However, after two years, both the seropositivity rates and GMC began to decline, dropping to 41.6% and 76.9 mIU/mL by the third year. Notably, from the fourth year onward, both the seropositivity rates and GMC increased again, reaching 49.2% and 109.6 mIU/mL, respectively. In contrast, children who received two doses of the varicella vaccine exhibited a rapid increase in seropositivity rates and GMC to 96.6% and 441.1 mIU/mL, respectively, within six months. Within one year, the seropositivity rates remained above 95%, while the GMC declined to 308.3 mIU/mL. After two years, both metrics continued to decrease, reaching 84.0% and 252.2 mIU/mL, respectively. After four years, these values further declined to 47.8% and 118.4 mIU/mL, respectively. In conclusion, despite the decline in antibody levels, within the four years following vaccination, the seropositivity rates and GMC of the population that received two doses of the varicella vaccine were significantly higher than those who received only one dose.

**Fig. 4:**
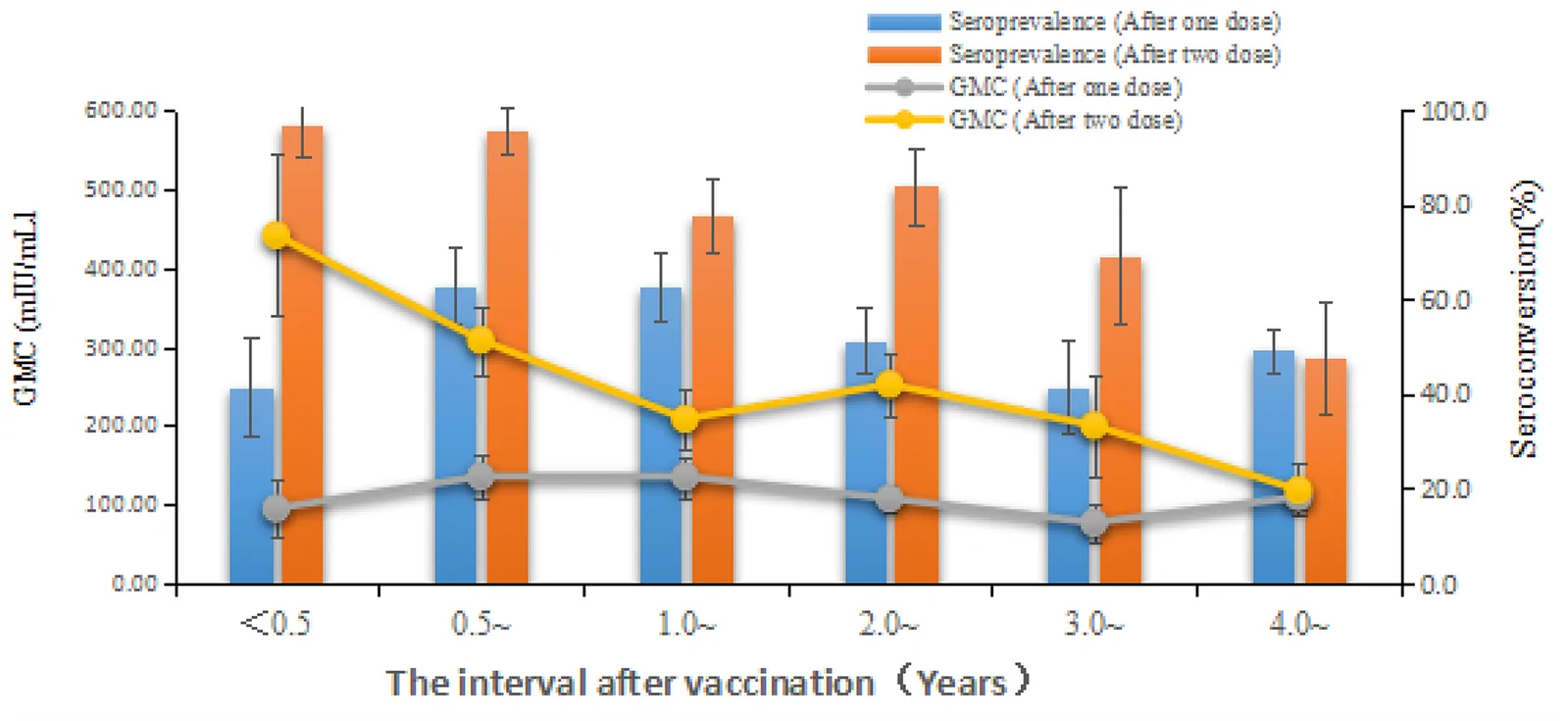
Changes in antibody levels at different time intervals after vaccination with different doses of varicella vaccine among 1–17 years old people in Fujian province. Error bars indicate 95% confidence intervals

## Discussions

In China, outbreaks of varicella have significantly disrupted schools and other child-centered institutions, resulting in a considerable disease burden on individuals and society [9]. In 2020, the incidence of varicella in Fujian Province decreased markedly, primarily due to stringent control measures implemented during the COVID-19 pandemic. Following 2021, the incidence of varicella exhibited a slight continuous decline, but began to rise again in 2024. Notably, even during the COVID-19 pandemic, the number of varicella-related outbreaks and cases remained relatively high. A total of 94 varicella outbreaks were reported in Fujian from 2019 to 2024, involving 461 cases, all of which occurred in schools. Varicella cases predominantly affected individuals aged 0–17 years, accounting for over 90% of total cases in Fujian Province, with a notable trend toward older age groups in recent years. For instance, the incidence in the 15–19 age group in 2024 was remarkable higher than in previous years, reaching 193.0 per 100,000. The prevalence of varicella is associated with various factors, including climate models, geographical locations, population density, exposure risks, and other potential influences [10]. To investigate the underlying causes of the high incidence of varicella among children and adolescents in Fujian Province, we analyzed changes in varicella vaccination rates and antibody levels among healthy individuals aged 0–17 years.

According to our survey results, the seropositivity rates and GMC of IgG antibodies against VZV in children aged 0–17 years in Fujian Province in 2023 were low, with values of only 47.7% and 81.3 mIU/mL, respectively. These figures are comparable to the results from Jiangsu Province in 2016, which reported a seropositivity rates of 43.5% [11]. However, they are significantly lower than those reported in Hangzhou City in 2018 (65.59%) [12] and in Beijing in 2012 (84.5%) [13]. This discrepancy may be attributed to the low coverage rate of the varicella vaccine in Fujian Province. Specifically, the coverage rate for individuals aged 1–17 years who received single dose of the varicella vaccine was 54.9%, while the rate for those receiving two doses was only 17.4%. These figures are markedly lower than those from a 2017 survey in Japan, which indicated a one-dose coverage rate of 67.0% among primary school students, with a two-dose coverage rate of 31.0% [14], which significantly less than the 80% threshold recommended by the WHO to achieve herd immunity [15]. In contrast, in Italy, a study assessing vaccination coverage in the 2019 birth cohort found that 92.08% of children were vaccinated by 24 months of age in 2021, largely due to mandatory vaccination policies [4]. Furthermore, several domestic cities, including Shanghai, Tianjin, and Suzhou, have incorporated the varicella vaccine into their local Expanded Program on Immunization (EPI) and are providing free vaccinations for children. The findings suggest that a higher vaccination rate correlates with improved protective effects for children [12, 15, 16].

Significant statistical differences in seropositivity rates and GMC were observed across different age groups. Children under one year old exhibited the lowest seropositive rate at 12.7%, with a GMC of 33.0 mIU/mL. These findings are consistent with research conducted in other provinces of China [13], potentially attributable to the gradual decline of maternal antibodies in this demographic and the timing of varicella vaccination [15]. As children age, those over one year old began receiving the varicella vaccine. Our survey indicated that 82.4% of individuals in Fujian Province who were willing to receive the varicella vaccine were vaccinated between the ages of one and two years. Consequently, the seropositive rate and GMC increased significantly to 49.5% and 86.6 mIU/mL, respectively, in children aged two years. Approximately 30% of children vaccinated between the ages of three and four years received a second dose of the vaccine, resulting in an increase in seropositivity rate and GMC among children aged three to five years. However, the low coverage of the second dose among school-aged children (17.4%) contributed to relatively minor fluctuations in seropositivity rate and GMC within this population, with seropositivity rates among individuals aged four to eleven years remaining between 43.9% and 53.6%. The GMC ranged from 91.8 mIU/mL to 115.4 mIU/mL. The persistence of low antibody levels is a significant factor contributing to the high incidence of varicella in this group [6]. Nonetheless, substantial statistical differences in seropositivity rates and GMC were still evident among individuals with varying vaccination histories, with the number of vaccine doses positively correlating with both seropositivity rates and GMC. This aligns with previous studies indicating that countries and provinces that implemented two doses of the varicella vaccine demonstrate enhanced protection compared to those administering a single dose [4, 16–18]. In terms of gender, the seropositivity rates and GMC of women were found to be higher than those of men. The basis of the sex difference in VZV seropositivity remains unclear and may involve the levels of virus exposure, incidence, or natural infection [12]. Females tend to have stronger innate and adaptive immune responses than males against both infections and vaccines [19], and males tend to engage in more activities and exhibit poorer health habits. Therefore, it is understandable that men are more susceptible to varicella [15].

To investigate the decline pattern of vaccine induced antibodies, we analyzed changes in antibody seropositivity rates and GMC over time after vaccination. Antibody levels remained elevated for 1–2 years following the initial dose; however, both seropositivity rates and GMC began to decline after the second year. By the third year, seropositivity rates and GMC had decreased by 33.8% and 43.4%, respectively. Notably, in the two-dose group, seropositivity rates and GMC increased rapidly to 96.6% and 441.1 mIU/mL within six months post‑vaccination, and remained high for one year. After one year, both values gradually decreased, and by the fourth year, they had declined by 50.5% and 73.2%, respectively. This pattern of antibody decline over time is generally consistent with other reports [11, 15]. For instance, Sun et al. [20] conducted a 42 month prospective study in Chinese children and observed that after a single dose, seropositivity rates remained 100% throughout the first 18 months but subsequently declined to 71.6% by 42 months, with GMC decreasing from 307.6 mIU/mL to 115.2 mIU/mL. However, our results differ significantly from the study by Prymula et al. [21], which evaluated the efficacy, immunogenicity, and safety of two doses of measles-mumps.

-rubella combined vaccine (MMRV) or one dose of monovalent varicella vaccine (V) in children from five Eastern European countries (Czech Republic, Lithuania, Poland, Romania, and Slovakia) over 10 years. Their results showed that at year 10, the seropositivity rate was 99.5–100% in the MMRV group and 98–100% in the MMR + V group, and the anti-VZV antibody GMCs were comparable between the two groups [21]. In contrast, our study found that in the two-dose group, although the seropositivity rate reached 96.6% within six months and remained high for one year, both the seropositivity rate and GMC declined substantially thereafter, decreasing by 50.5% and 73.2%, respectively, by year four. This discrepancy underscores the potential influence of several factors on antibody kinetics, including vaccine type, vaccination interval, population background (including baseline VZV exposure levels), analytical methodology, laboratory detection techniques, and follow-up duration [20, 21].

In addition, in this study, the seropositive rate and GMC decreased markedly after one year of vaccination regardless of whether the first dose or two doses were given, but the decline rate was steeper in the two-dose group. This difference may reflect distinct antibody kinetics, with a slower decrease in the basal titer after a single dose despite a lower basal titer. Although the initial immune response is stronger after two doses, the rapid declining trend of antibodies cannot be ignored. A study on persistence of immunity after two doses of varicella vaccine in Chinese children reported a notable decrease in both seroprevalence and GMC over time, with seroprevalence decreasing from 100.0% to 79.8% and GMC decreasing from 575.1 mIU/mL to 251.9 mIU/mL [22]. Furthermore, a retrospective cohort study of fully immunized health care workers in Italy reported that 34% of individuals who received two doses of varicella vaccine lacked protective antibody titers at follow-up, although the third dose restored protective antibody titers in all cases [23]. This finding further emphasizes that while two doses provide better initial protection than one dose, antibody decay remains a concern even in fully immunized adults, and additional booster doses may be necessary for certain high-risk groups.

Several limitations of this study should be acknowledged. Firstly, this study did not fully consider the participants’ history of varicella infection, whether in the children themselves or their family members, which may have influenced the baseline immune status. Secondly, calculating vaccine coverage only for individuals with vaccination records, while treating those without records as unvaccinated, may underestimate the true population coverage rate. Thirdly, this study is essentially a cross-cohort comparison at different time points rather than a longitudinal follow-up. Therefore, the “antibody waning” trend observed across different time intervals is subject to potential bias. Fourth, due to limitations of the ELISA reagents used, the antibody levels induced by the virus may be underestimated. However, these limitations do not affect the analysis of the overall antibody distribution among the young population in Fujian Province, nor do they prevent the identification of susceptible populations.

## Conclusions

Finally, varicella antibody levels were low and vaccination coverage was suboptimal among individuals aged 0–17 years in Fujian Province. The seropositivity rate of vaccine-induced antibodies declined significantly over time. Notably, T-cell-mediated immune memory can be maintained for a prolonged period after vaccination; thus, even if humoral antibody levels decrease, some degree of clinical protection may still be conferred [24]. Given the high incidence of varicella, continued strengthening of varicella antibody surveillance in the population is warranted. Optimizing the timing of the two-dose regimen should be considered a key direction for future research.

## Data Availability

The datasets generated and/or analyzed during the current study are available from the corresponding author on reasonable request.
